# Progressive Bilateral Weakness Followed by Unilateral Facial Palsy in Guillain-Barré Syndrome Temporally Associated With Multiple Vaccinations

**DOI:** 10.7759/cureus.116823

**Published:** 2026-09-24

**Authors:** Kian Memari, Daniela Prado Escobar, Sergio A Rodriguez, Ellison Marlar, Joren Manuel, Peter Cohen, Shane Williams, Lissette P Lazo

**Affiliations:** 1 Family Medicine, Palmetto General Hospital, Hialeah, USA; 2 Internal Medicine, Nova Southeastern University Dr. Kiran C. Patel College of Osteopathic Medicine, Clearwater, USA; 3 Family Medicine, Nova Southeastern University Dr. Kiran C. Patel College of Osteopathic Medicine, Fort Lauderdale, USA

**Keywords:** albuminocytologic dissociation, bell’s palsy, diagnostic anchoring, facial nerve palsy, guillain-barré syndrome, intravenous immunoglobulin, paresthesias, vaccination

## Abstract

Guillain-Barré syndrome (GBS) is an acute immune-mediated polyradiculoneuropathy in which facial weakness may occur as a cranial neuropathy. Unilateral facial palsy may also prompt a diagnosis of idiopathic Bell’s palsy, creating a risk of diagnostic anchoring when broader neurologic symptoms are already present. We report a 32-year-old man with childhood epilepsy who described progressive bilateral upper and lower extremity weakness with paresthesias for approximately two weeks, followed by left facial paralysis about one week later. He also reported co-administration of hepatitis B, influenza, and tetanus vaccines approximately three weeks before presentation; the vaccine products and dates were not independently verified. An outside hospital treated the facial paralysis as presumed Bell’s palsy with acyclovir, although the available records do not establish the facial examination pattern, acyclovir regimen, whether corticosteroids were prescribed there, or whether the preceding bilateral limb symptoms were recognized. An outpatient electrodiagnostic study was reportedly interpreted as concerning for GBS, but the complete nerve conduction/electromyography report was unavailable. Cerebrospinal fluid (CSF) showed white blood cells of 5/mm³, glucose of 59 mg/dL, and protein of 190 mg/dL, supporting albuminocytologic dissociation; a paired serum glucose was unavailable. The patient completed a five-day course of intravenous immunoglobulin (IVIG), and prednisone plus valacyclovir was also documented for the facial palsy, although their exact dose, route, and duration were not recoverable. The source record documented short-term neurologic improvement before discharge. This case emphasizes that unilateral facial palsy should not distract from pre-existing bilateral weakness and paresthesias and that facial weakness in this setting may represent GBS-related cranial neuropathy rather than a separate confirmed Bell’s palsy. The temporal association with vaccination does not establish causality.

## Introduction

Guillain-Barré syndrome (GBS) is an acute immune-mediated polyradiculoneuropathy with an estimated incidence of approximately 1-2 cases per 100,000 person-years [1]. The classic syndrome consists of rapidly progressive, relatively symmetric limb weakness, sensory symptoms, and diminished or absent reflexes, with variable cranial nerve, bulbar, autonomic, and respiratory involvement [2]. Because clinical deterioration can occur over hours to days, timely recognition, respiratory surveillance, and appropriate disease-modifying treatment are essential.

GBS most often follows an infection. Molecular mimicry is a leading proposed mechanism in which antibodies generated against an antecedent immune stimulus cross-react with peripheral nerve components, producing demyelinating or axonal injury [3]. Immunization has been reported as an uncommon antecedent event, particularly after influenza vaccination, but the absolute risk is very low, and causality is difficult to establish in an individual patient [4]. Accordingly, a case occurring after vaccination should be described as temporally associated unless stronger epidemiologic or mechanistic evidence supports causation.

Facial weakness is a recognized cranial neuropathy in GBS. Contemporary diagnostic frameworks particularly recognize bilateral facial weakness as a supportive feature; unilateral facial palsy is less typical and may be confused with idiopathic Bell’s palsy [2,5]. Bell’s palsy is an acute unilateral lower motor neuron facial palsy involving the peripheral seventh cranial nerve and is a diagnosis of exclusion. Early corticosteroid therapy improves the probability of complete recovery, while the incremental benefit of antiviral therapy is less certain [6,7]. When facial weakness occurs in a patient who already has bilateral limb weakness or paresthesias, the broader neurologic syndrome should take priority over anchoring on an isolated facial nerve diagnosis.

The diagnosis of GBS remains primarily clinical and may be supported by electrodiagnostic testing and cerebrospinal fluid (CSF) analysis. Albuminocytologic dissociation, defined by elevated CSF protein without pleocytosis, is a classic supportive finding [5]. Intravenous immunoglobulin (IVIG) and plasma exchange are established disease-modifying treatments, and current guidance emphasizes serial assessment for respiratory, bulbar, and autonomic deterioration [8]. We present a case in which bilateral limb symptoms preceded unilateral facial paralysis, emphasizing a diagnostic anchoring pitfall rather than a sequence in which facial palsy preceded or “foreshadowed” generalized weakness.

## Case presentation

A 32-year-old man with a history of childhood epilepsy presented to the emergency department after an outpatient electromyography and nerve conduction evaluation was reportedly interpreted as concerning for GBS. The complete electrodiagnostic report was not available in the reviewed source documentation. Consequently, motor and sensory amplitudes, conduction velocities, distal latencies, F-wave responses, conduction block, temporal dispersion, needle electromyography findings, and a specific electrophysiologic subtype could not be independently assessed.

The patient reported approximately two weeks of progressive bilateral upper and lower extremity weakness with paresthesias. Left facial paralysis began approximately one week before admission, meaning that the limb symptoms preceded the facial symptoms by about one week. He had recently immigrated from Cuba and reported receiving hepatitis B, influenza, and tetanus vaccines during a single vaccination encounter approximately three weeks before presentation. The vaccination dates and exact products were based on patient recollection and were not independently verified.

Approximately one week before admission, he was evaluated at an outside hospital for the left facial paralysis and was prescribed acyclovir for presumed Bell’s palsy. The available source record does not provide the acyclovir dose, route, planned duration, or exact interval from facial symptom onset to treatment. It also does not establish whether corticosteroids were prescribed at the outside hospital, whether the pre-existing bilateral limb weakness and paresthesias were elicited at that visit, or whether a broader neurologic examination was performed. These omissions prevent a reliable reconstruction of the outside hospital diagnostic process.

At the subsequent hospital evaluation, the record documented left-sided facial paralysis and clinically significant generalized weakness, but it did not preserve the facial examination details necessary to classify the palsy as central or peripheral. Specifically, forehead involvement, eye closure strength, the degree of lower facial weakness, and other seventh nerve examination findings were unavailable. Likewise, formal Medical Research Council strength grades, deep tendon reflex scores, a detailed sensory map, and gait findings were not available. Therefore, idiopathic Bell’s palsy could not be independently confirmed, and GBS-related facial nerve involvement remained a plausible alternative.

The patient denied fever, weight loss, night sweats, known sick contacts, headache, visual symptoms, nausea, vomiting, diarrhea, stool changes, abdominal pain, chest pain, dyspnea, and other systemic symptoms. The bilateral distribution and progression of the limb symptoms were not explained by an isolated facial nerve palsy and prompted evaluation for an acute peripheral polyradiculoneuropathy (Table 1).​​

**Table 1:** Source-documented clinical and diagnostic findings EMG: electromyography, NCS: nerve conduction study, CSF: cerebrospinal fluid, CT: computed tomography, IVIG: intravenous immunoglobulin

Domain	Documented finding	Interpretive significance/limitation
Chronology	Bilateral arm and leg weakness with paresthesia for approximately two weeks; left facial paralysis for approximately one week	Limb symptoms preceded facial paralysis by about one week
Facial weakness	Left facial paralysis documented; forehead, eye closure, and lower-face distribution not preserved in the available record	Central versus peripheral pattern cannot be independently classified; Bell’s palsy remains an initial working diagnosis rather than a confirmed concurrent disorder
Vaccination history	Patient-reported hepatitis B, influenza, and tetanus vaccine co-administration approximately three weeks before presentation	Temporal association only; dates/products were not independently verified
Electrodiagnostic testing	Outpatient EMG/NCS reportedly interpreted as concerning for GBS; complete report unavailable	Prompted urgent hospital evaluation but does not permit independent confirmation of electrophysiologic criteria or subtype
CSF white blood cells	5 cells/mm³ (reference range: 0-5 cells/mm³)	No pleocytosis
CSF glucose	59 mg/dL (reference range: 40-70 mg/dL)	Within the laboratory range; paired serum glucose was unavailable, so a CSF-to-serum glucose ratio cannot be calculated
CSF total protein	190 mg/dL (reference range: 15-45 mg/dL)	Markedly elevated; with no pleocytosis, supports albuminocytologic dissociation
Head CT	No acute intracranial hemorrhage, mass effect, or other acute structural abnormality	Contextual evaluation for central structural pathology; does not diagnose or exclude a peripheral facial neuropathy
Treatment	Five-day IVIG course; inpatient prednisone and valacyclovir also documented	IVIG was directed at suspected GBS; exact IVIG daily dose and exact prednisone/valacyclovir dose, route, duration, and timing were not recoverable
Outcome	Source record documented resolution of neurologic symptoms before discharge	Favorable short-term course; objective serial neurologic scores and long-term follow-up were unavailable

An initial lumbar puncture in the emergency department was unsuccessful. Because of progressive bilateral symptoms and the reported outpatient electrodiagnostic concern for GBS, IVIG was initiated without waiting for CSF confirmation, and the patient was admitted to a critical care setting for close neurologic and respiratory observation. Neurology was consulted.

A subsequent lumbar puncture produced clear, colorless CSF with a white blood cell count of 5 cells/mm³ (reference range: 0-5 cells/mm³), glucose of 59 mg/dL (reference range: 40-70 mg/dL), and total protein of 190 mg/dL (reference range: 15-45 mg/dL). The markedly elevated protein in the absence of pleocytosis supported albuminocytologic dissociation. A paired serum glucose obtained at or near the lumbar puncture was not available in the source record; therefore, the CSF-to-serum glucose ratio could not be calculated, and the CSF glucose is reported only relative to the laboratory reference range.

Chest radiography was unremarkable. Non-contrast head CT showed no acute intracranial hemorrhage, mass effect, or other acute structural abnormality on sagittal view (Figure 1) and axial view (Figure 2). These images were contextual in the evaluation of a central structural process and did not establish GBS or Bell’s palsy.

**Figure 1:**
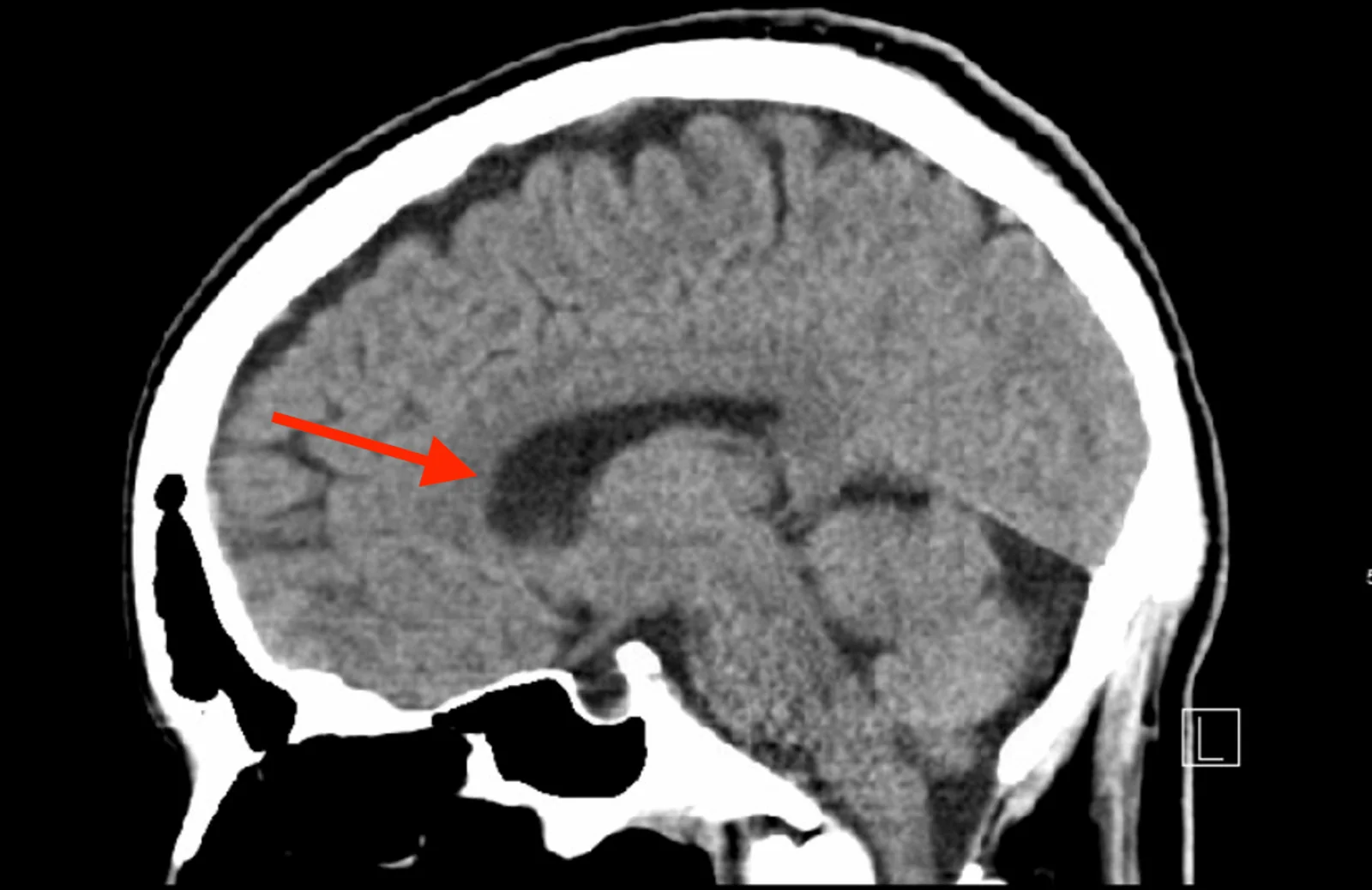
Non-contrast head CT (sagittal view) Sagittal non-contrast head CT demonstrating no acute intracranial mass effect or midline shift. The red arrow identifies the lateral ventricle. The study was contextual in excluding a major acute structural intracranial process and does not diagnose a peripheral facial neuropathy or GBS. CT: computed tomography, GBS: Guillain-Barré syndrome

**Figure 2:**
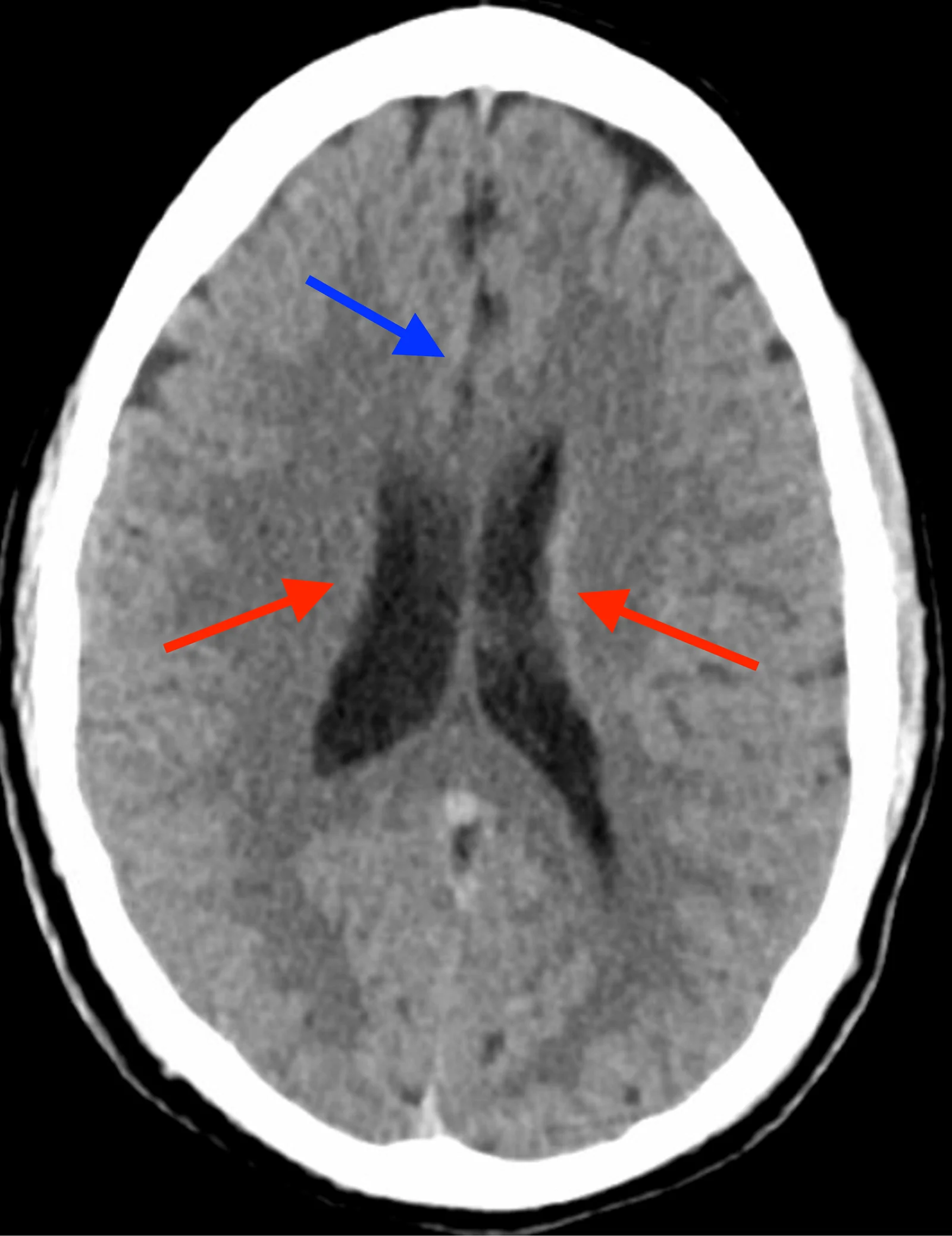
Non-contrast head CT (axial view) Axial non-contrast head CT demonstrating symmetric lateral ventricles (red arrows) and a preserved midline (blue arrow), without acute hemorrhage or mass effect. The image is contextual and is not diagnostic of GBS or Bell’s palsy. CT: computed tomography, GBS: Guillain-Barré syndrome

The patient completed a five-day course of IVIG. The source record does not preserve the patient’s weight-based daily IVIG dose or exact infusion times. Prednisone and valacyclovir were also documented during the hospitalization because presumed Bell’s palsy remained in the differential diagnosis; however, their exact dose, route, duration, and timing relative to IVIG were not recoverable from the available records. The record does not support attributing neurologic improvement to any single therapy. It documented resolution of the neurologic symptoms before discharge, no respiratory decompensation requiring mechanical ventilation, and no documented treatment-limiting adverse effect. Detailed serial respiratory mechanics, autonomic data, objective pre- and post-treatment neurologic scores, and long-term neurologic follow-up were unavailable (Table 2).

**Table 2:** Clinical timeline preserving the documented sequence of events ED: emergency department, LP: lumbar puncture, GBS: Guillain-Barré syndrome, IVIG: intravenous immunoglobulin, CSF: cerebrospinal fluid, WBC: white blood cell count

Time point	Clinical event
~3 weeks before presentation	Patient-reported co-administration of hepatitis B, influenza, and tetanus vaccines in Cuba; documentation of exact products/dates unavailable
~2 weeks before presentation	Progressive bilateral upper and lower extremity weakness with paresthesia begins
~1 week before presentation	Left facial paralysis develops after limb symptoms had already begun; outside hospital diagnoses presumed Bell’s palsy and prescribes acyclovir; detailed examination and treatment regimen unavailable
Hospital day 0	Outpatient electrodiagnostic study is reportedly interpreted as concerning for GBS, prompting ED referral; initial LP unsuccessful; IVIG initiated, and patient admitted to critical care for neurologic/respiratory observation
Hospital day 1	Successful LP shows CSF protein of 190 mg/dL with WBC of 5 cells/mm³, supporting albuminocytologic dissociation; neurology involved
During hospitalization	Five-day IVIG course completed; prednisone and valacyclovir also documented for presumed Bell’s palsy; exact regimen not recoverable
Discharge	Source record documents resolution of neurologic symptoms; objective serial examination metrics and longer-term outcome unavailable

## Discussion

This case illustrates a potential diagnostic anchoring pitfall: unilateral facial palsy arising after bilateral limb symptoms may divert attention from an evolving generalized neurologic syndrome. The documented chronology is clear: bilateral arm and leg weakness with paresthesias began approximately two weeks before admission, whereas unilateral facial paralysis began approximately one week before admission. Thus, the facial palsy occurred during an already evolving generalized neurologic syndrome. When it was assessed as presumed Bell’s palsy at the outside hospital, the pre-existing bilateral limb symptoms, if present and elicited at that encounter, should have prompted consideration of a broader peripheral neuropathic process.

Facial nerve involvement is well recognized in GBS, with bilateral facial weakness being the more characteristic pattern highlighted in contemporary diagnostic frameworks [2,5]. Unilateral facial palsy is less typical and may resemble idiopathic Bell’s palsy. The present record does not document forehead involvement, eye closure weakness, lower facial distribution, or other findings required to establish a peripheral seventh nerve pattern. Accordingly, the available documentation does not establish Bell’s palsy as a confirmed concurrent diagnosis. The most defensible formulation is that Bell’s palsy was an initial working diagnosis, whereas facial nerve involvement within the GBS spectrum remained a plausible explanation once the bilateral limb syndrome was recognized.

The available diagnostic evidence supports GBS but does not allow retrospective confirmation of a specific electrodiagnostic subtype or a formal diagnostic certainty level. The outpatient electrodiagnostic study was reportedly considered concerning for GBS, but the complete report was unavailable, including amplitudes, conduction velocities, distal latencies, F-wave data, conduction block, temporal dispersion, and needle electromyography findings. Therefore, acute inflammatory demyelinating polyradiculoneuropathy could not be confirmed. CSF protein of 190 mg/dL with white blood cells of 5/mm³ provided independent supportive evidence of albuminocytologic dissociation, although this finding is not specific to GBS [5].

The treatment record also requires cautious interpretation. Current GBS guidance supports IVIG or plasma exchange as disease-modifying therapy; a commonly recommended IVIG regimen is 0.4 g/kg/day for five days in patients meeting treatment criteria [8]. The available source confirms that this patient completed a five-day IVIG course but does not preserve the exact daily dose. Corticosteroids are not recommended as treatment for GBS [8]. By contrast, early corticosteroid therapy improves outcomes in idiopathic Bell’s palsy [6,7]. The outside hospital record available to us documents acyclovir for presumed Bell’s palsy but does not establish whether a corticosteroid was prescribed there. Prednisone and valacyclovir were documented during the subsequent hospitalization, but their exact regimen and timing were unavailable. Because the exact temporal relationship among IVIG, prednisone, and valacyclovir was unavailable, the observed short-term improvement cannot be attributed to a single intervention.

The vaccination history should likewise be interpreted conservatively. The patient recalled hepatitis B, influenza, and tetanus vaccines administered during one encounter approximately three weeks before presentation, placing symptom onset within a biologically plausible interval for an immune-mediated event. However, the vaccine products and dates were not independently verified; infection is a much more common antecedent to GBS, and a single case cannot establish causality or identify one vaccine as responsible [3,4]. We therefore describe vaccination only as a patient-reported temporal association.

The central teaching point is practical: facial paralysis should not distract from neurologic symptoms that predate it. In a patient with unilateral facial weakness, clinicians should actively ask about bilateral limb weakness, paresthesias, progression, gait change, bulbar symptoms, dyspnea, and autonomic symptoms, and should perform a complete motor, sensory, reflex, cranial nerve, gait, respiratory, and autonomic assessment when GBS is possible. These are general warning features and recommended elements of evaluation; several of them were not documented in the source record for this patient and are not presented here as case-specific findings.

This report has important limitations. The facial examination pattern, formal limb strength and reflex grading, detailed sensory and gait examination, full electrodiagnostic report, paired serum glucose, exact IVIG dose, complete acyclovir/prednisone/valacyclovir regimens, serial respiratory mechanics, autonomic measurements, independently verified vaccine records, and long-term neurologic follow-up were unavailable. These gaps preclude confirmation of idiopathic Bell’s palsy as a separate diagnosis, formal electrophysiologic subtyping, calculation of a CSF-to-serum glucose ratio, attribution of recovery to a single therapy, and strong causal inference regarding vaccination. Interpretation is therefore limited to the source-supported observations and their diagnostic implications.

## Conclusions

In this patient, progressive bilateral upper and lower extremity weakness with paresthesias preceded unilateral facial paralysis by approximately one week. The later facial palsy was initially treated as presumed Bell’s palsy, but the available documentation does not establish a peripheral facial nerve examination pattern, and GBS-related facial nerve involvement remained a plausible explanation. The broader bilateral neurologic syndrome, an outpatient electrodiagnostic study reportedly concerning for GBS, and CSF albuminocytologic dissociation supported the diagnosis of GBS, while the unavailable full electrodiagnostic report precluded subtype confirmation.

The clinical lesson is to avoid anchoring on facial palsy when bilateral limb symptoms are already present and to pursue a complete neurologic and respiratory assessment when GBS is possible. Short-term improvement was documented during a hospitalization in which IVIG and facial palsy-directed therapies were administered, so treatment-specific attribution is not possible. The reported recent vaccination history represents temporal association only and does not establish causality.
